# An emotion recognition method based on frequency-domain features of PPG

**DOI:** 10.3389/fphys.2025.1486763

**Published:** 2025-02-25

**Authors:** Zhibin Zhu, Xuanyi Wang, Yifei Xu, Wanlin Chen, Jing Zheng, Shulin Chen, Hang Chen

**Affiliations:** ^1^ College of Biomedical Engineering and Instrument Science, Zhejiang University, Hangzhou, China; ^2^ Department of Psychology and Behaviorial Sciences, Zhejiang University, Hangzhou, China; ^3^ Zhejiang Provincial Key Laboratory of Cardio-Cerebral Vascular Detection Technology and Medicinal Effectiveness Appraisal, Hangzhou, China; ^4^ Connected Healthcare Big Data Research Center, Zhejiang Lab, Hangzhou, China

**Keywords:** photoplethysmography (PPG), emotion recognition, support vector machine (SVM), PPG frequency-domian analysis, dual windkessel model

## Abstract

**Objective:**

This study aims to employ physiological model simulation to systematically analyze the frequency-domain components of PPG signals and extract their key features. The efficacy of these frequency-domain features in effectively distinguishing emotional states will also be investigated.

**Methods:**

A dual windkessel model was employed to analyze PPG signal frequency components and extract distinctive features. Experimental data collection encompassed both physiological (PPG) and psychological measurements, with subsequent analysis involving distribution patterns and statistical testing (U-tests) to examine feature-emotion relationships. The study implemented support vector machine (SVM) classification to evaluate feature effectiveness, complemented by comparative analysis using pulse rate variability (PRV) features, morphological features, and the DEAP dataset.

**Results:**

The results demonstrate significant differentiation in PPG frequency-domain feature responses to arousal and valence variations, achieving classification accuracies of 87.5% and 81.4%, respectively. Validation on the DEAP dataset yielded consistent patterns with accuracies of 73.5% (arousal) and 71.5% (valence). Feature fusion incorporating the proposed frequency-domain features enhanced classification performance, surpassing 90% accuracy.

**Conclusion:**

This study uses physiological modeling to analyze PPG signal frequency components and extract key features. We evaluate their effectiveness in emotion recognition and reveal relationships among physiological parameters, frequency features, and emotional states.

**Significance:**

These findings advance understanding of emotion recognition mechanisms and provide a foundation for future research.

## 1 Introduction

Emotions represent a complex array of psychological and physiological reactions that individuals experience in response to specific stimuli (Shu et al., 2018). The spectrum of emotional states can exert varying influences on an individual’s physical and mental wellbeing, potentially precipitating severe health conditions (Ong et al., 2006) For instance, chronic exposure to negative emotional states has been linked to the etiology of mood disorders such as depression and anxiety (Button et al., 2012; Gou et al., 2023). Consequently, the accurate identification of emotions has emerged as a pivotal area of inquiry within the realm of psychological research.

The method of emotion assessment based on physiological signals stands out for its capability to collect data autonomously and discern emotional states (Maria et al., 2019), offering a significant advantage over traditional approaches that rely on subjective emotional scales (Bradley and Lang, 1994) and physical cues (Anthony and Patil, 2023; Harms et al., 2010; Hasan et al., 2019). Unlike these, physiological signals, which are inherently spontaneous and less prone to subjective influences, provide a more objective measure of emotional responses (Jang et al., 2014). The activation of emotions is inherently linked to the central nervous system’s regulatory functions. This has prompted a multitude of studies to extract multifaceted features from electroencephalogram (EEG) signals (Alvarez-Jimenez et al., 2024; Issa et al., 2021; Li et al., 2022; Sarma and Barma, 2021), aiming to construct models for emotion identification or to investigate effective methods through the application of deep learning algorithms (Dhara et al., 2023; Joshi and Ghongade, 2021). Beyond EEG, the realm of emotion identification research has also incorporated a range of other physiological signals. These include electrocardiogram (ECG) (Hsu et al., 2020; Sarkar et al., 2022), which captures the heart’s electrical activity; electromyography (EMG) (Kulke et al., 2020; Sato et al., 2008), which measures muscle electrical activity; and galvanic skin response (GSR) (Goshvarpour et al., 2017; Wen et al., 2014), which reflects the body’s sweat gland activity in response to emotional stimuli. Each of these modalities contributes unique insights into the complex interplay between physiological responses and emotional experiences.

The burgeoning ubiquity of portable devices has catapulted photoplethysmography (PPG) into the spotlight of research communities, thanks to its notable benefits such as ease of acquisition, operational simplicity, and minimal equipment costs. Concurrently, the existing body of research has established that a plethora of physiological changes triggered by emotional stimuli are modulated by the autonomic nervous system (ANS) (Gordan et al., 2015; Rainville et al., 2006), impacting vital organs like the heart, blood vessels, and muscles (Harris and Matthews, 2004; Kleiger et al., 2005). These physiological shifts are vividly reflected in PPG signals, serving as a tangible indicator of the body’s response to emotions (Chakraborty et al., 2020). For instance, the emotion of fear can induce vasoconstriction and tachycardia (Johnstone, 1971), while anger may lead to vasodilation in facial blood vessels, resulting in blushing and arrhythmia (Drummond, 1999). The spectrum of human emotions elicits a diverse array of effects on PPG signals (Davydov et al., 2011; Krumhansl, 1997), each offering a unique perspective on the intricate relationship between emotional states and physiological responses (Nummenmaa et al., 2014).

To date, the body of research leveraging photoplethysmography (PPG) signals for precise emotion recognition remains modest. Notable contributions include Paul’s work (Paul et al., 2024), where a novel time-domain feature was extracted from the DEAP (Koelstra et al., 2012) dataset to discern various emotional states. Beckmann et al. (2019), in another study, employed dual sensors to capture PPG’s Perfusion Time to Peak (PTT) features, subsequently integrating them into the realm of wearable device-based emotion recognition research. Li et al. (2017) contributed by acquiring both PPG morphological and PRV features to differentiate between states of sadness and happiness. Furthermore, Wang and Yu (2021) and Lee et al. (2020) have ventured into the application of deep learning methodologies for the analysis of PPG signals in emotion recognition, showcasing the potential of these advanced techniques.

Previous studies have made significant progress in the field of emotion recognition using PPG signals, yet further exploration remains warranted. Current research predominantly focuses on PRV and morphological features, with limited exploration of frequency-domain analysis of PPG waveforms. Given that the frequency domain of signals often contains substantial valuable information, this study aims to employ physiological model simulation to systematically analyze the frequency-domain components of PPG signals and extract their key features. Furthermore, this research will investigate the efficacy of these frequency-domain features in effectively distinguishing emotional states, thereby contributing to the advancement of emotion recognition methodologies.

## 2 Methods

**FIGURE 1:**
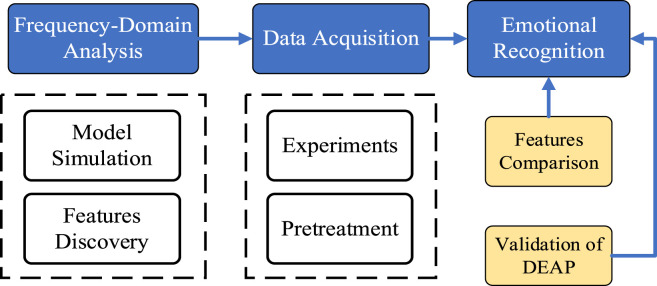
The schematic diagram of the emotion recognition method.

The schematic diagram presented in Figure 1 delineates the emotion recognition methodology predicated on frequency-domain features derived from photoplethysmography (PPG) signals. The process encompasses several pivotal steps: 1) Constructing physiological simulation models, conducting frequency domain analysis, and extracting key features; 2) Designing experiments to collect PPG signals and preprocessing them; 3) Accurate recognition of emotional states; 4) Extracting PRV and morphological features for comparison and finally 5) Verifying the recognition universality based on PPG signals collected from the DEAP dataset.

### 2.1 Frequency domain analysis based on simulated physiological model

**FIGURE 2:**
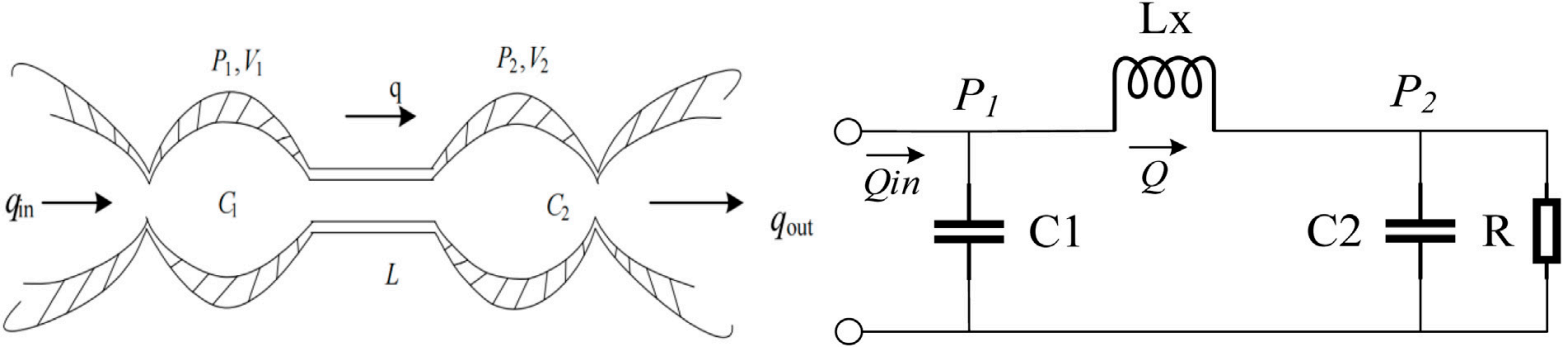
**(A)** The dual windkessel model. **(B)** The equivalent circuit of the dual windkessel model.

Consequently, the development of PPG simulation models is of paramount importance for investigating the influence of different physiological factors on PPG signals. Among the available cardiovascular system models, the dual windkessel model Goldwyn and Watt (1967) proposed by Goldwyn and Watt stands out as one of the most frequently utilized frameworks. This model, along with its equivalent circuit representation, is depicted in Figure 2, providing a visual and theoretical foundation for understanding the complex dynamics of the cardiovascular system as they relate to PPG signal generation. This study constructed a simulation design for Simulink based on this model and obtained 7 variable parameters as shown in Table 1.

**TABLE 1:** The 7 variable parameters of the model.

Parameters	Description
*R*	The magnitude of peripheral vascular resistance
*L*	The magnitude of blood flow inertia
*C*_*1*_	Aggregate compliance of the aortic arch and its main branches
*C*_*2*_	Total compliance of aorta and peripheral blood vessels
*Q*_*0*_	Extreme point of blood flow
*Ts*	The duration of systole
*Td*	The duration of the heartbeat cycle

**FIGURE 3:**
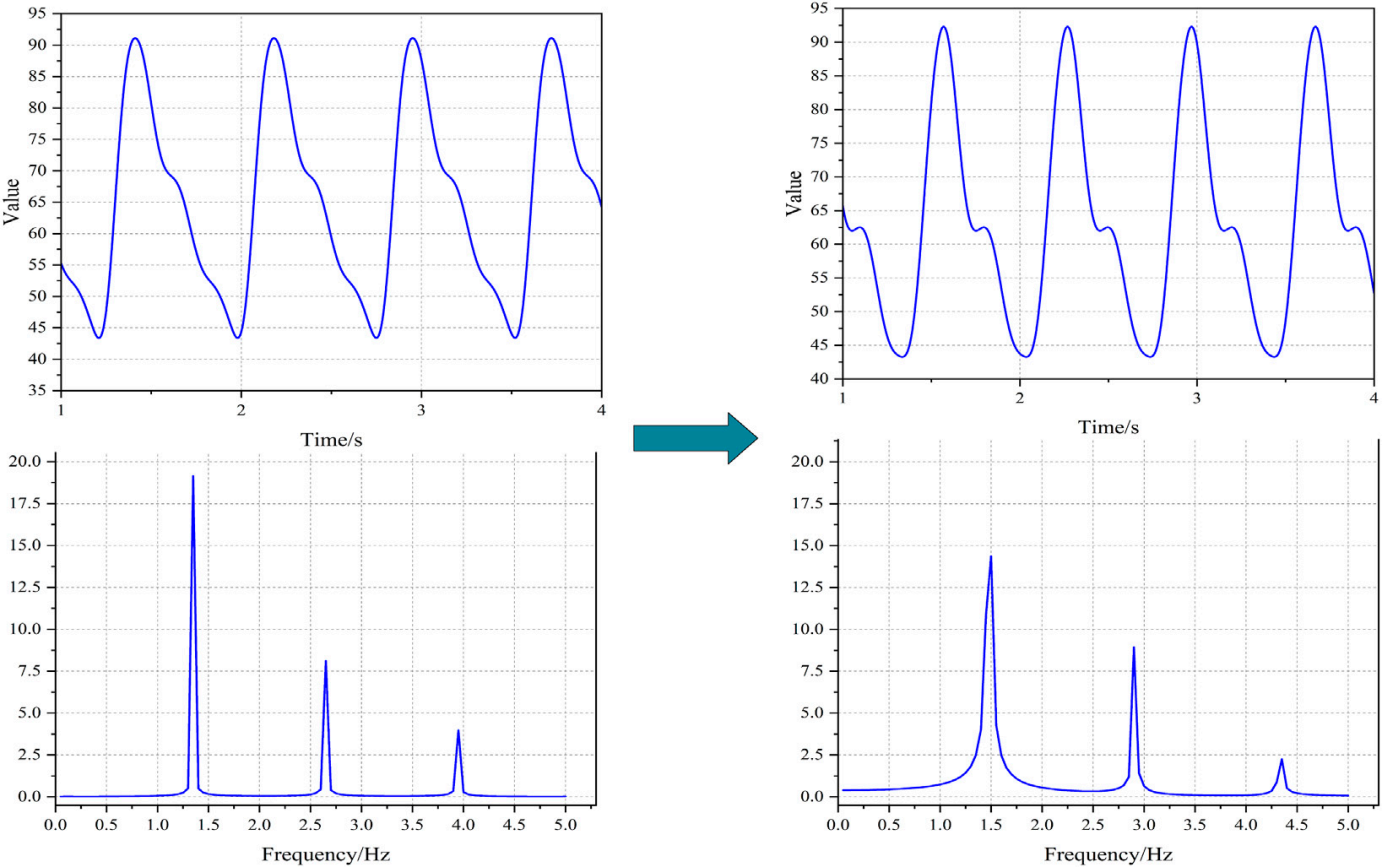
The PPGs under different states based on simulation. Notes: The physiological parameters were configured as follows: Left-side: R = 0.8, C₁ = 0.8, C₂ = 0.18, L = 0.008, T_d_ = 0.77, T_s_ = 0.28, Q₀ = 395; Right-side: R = 1.2, C₁ = 1.2, C₂ = 0.20, L = 0.012, T_d_ = 0.67, T_s_ = 0.25, Q₀ = 450.

By systematically modulating the aforementioned variable parameters, significant alterations were observed in both the simulated PPG waveforms and their corresponding frequency spectra. As illustrated in Figure 3, the morphological state of the PPG waveforms exhibited visually discernible variations. Furthermore, distinct differences were identified in the spectral peaks of the frequency domain representation, demonstrating the sensitivity of these spectral components to parameter variations.

**FIGURE 4:**
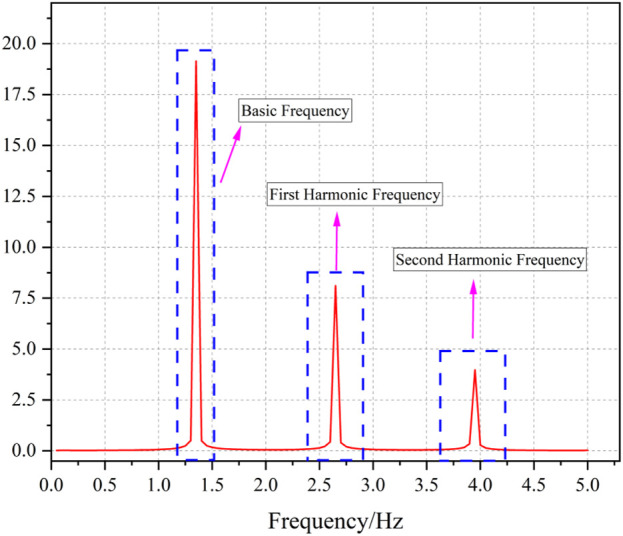
Frequency domain information of PPG.

Our analysis reveals that the frequency-domain information of the photoplethysmography (PPG) signal is predominantly characterized by its fundamental frequency and two harmonic frequency bands. Consequently, we derived multiple power-related features and ratio-based features from these three frequency bands as shown in Figure 4. The specific features and their corresponding variations in response to parameter changes during the simulation are comprehensively presented in Table 2. The computation of these features was performed through the following procedure: First, the average heart rate (HR) was obtained through preliminary data processing. Subsequently, Fast Fourier Transform (FFT) analysis was applied to the data segment. The power spectral density within the frequency bands of ±0.2 Hz centered at the fundamental HR frequency was identified as the Basic Frequency component (BF). Similarly, the power within ±0.2 Hz bands centered at twice and three times the HR frequency were designated as the First Harmonic Frequency (FHF) and Second Harmonic Frequency (SHF) components, respectively. The computational methodology for these ratio-based features is further detailed in Table 2.

**TABLE 2:** Description of PPG frequency domain features.

Features	Description	*R ↑*	*C1 ↑*	*C2 ↑*	*L ↑*	*Q0 ↑*	*Td ↑*	*Ts ↑*
BF	Power of the base frequency band	**↓**	**↓**	**↓**	**↑**	**↑**	**↑**	**↑**
BFn	Standardized power of the base frequency band, BF/(BF + FHF + SHF)	**↓**	**↓**	**↓**	**↓**	**↑**	**↑**	**↑**
FHF	Power of the first harmonic frequency band	**↑**	**↓**	**↑**	**↑**	**↑**	**↑**	**↓**
FHFn	Standardized power of the first harmonic frequency band, FHF/(BF + FHF + SHF)	**↑**	**↑**	**↑**	**↑**	**0**	**↓**	**↓**
SHF	Power of the second harmonic frequency band	**↑**	**↓**	**↓**	**↓**	**0**	**↑**	**↓**
SHFn	Standardized power of the second harmonic frequency band, SHF/(BF + FHF + SHF)	**↑**	**↓**	**↓**	**↓**	**0**	**↑**	**↓**
FHFBF	FHF/BF	**↑**	**↑**	**↑**	**↑**	**0**	**↓**	**↓**
SHFBF	SHF/BF	**↑**	**↓**	**↓**	**↓**	**0**	**↑**	**↓**
SHFFHF	SHF/FHF	**↑**	**↓**	**↓**	**↓**	**0**	**↑**	**↓**

The analysis demonstrates distinct patterns in frequency features corresponding to variations in hemodynamic parameters: (1) Increased peripheral vascular resistance leads to attenuation of the fundamental frequency while enhancing harmonic frequencies. (2) Elevated vascular compliance results in amplification of the first harmonic frequency, accompanied by attenuation of both the fundamental frequency and the second harmonic frequency. (3) Augmented blood flow inertia induces enhancement of both the fundamental frequency and the first harmonic frequency, with the latter exhibiting more pronounced amplification, while simultaneously causing attenuation of the second harmonic frequency. Furthermore, other physiological parameters also exert significant influences on these features.

### 2.2 Dataset and preprocessing

This dataset is anchored in Ekman and Friesen (1971) theory of discrete emotions, which posits that emotions are distinct, universally identified mental states. In alignment with this theoretical framework and to ensure the selection of authentic and impactful emotion-inducing materials, our research team referred to authoritative emotion databases. Notably, we took cues from the DECAF database (Abadi et al., 2015) in curating a selection of video materials designed to elicit a variety of emotional responses. Our team has conducted extensive prior research to thoroughly assess and validate the efficacy of these selected materials in inducing the intended emotions during emotion induction experiments (Wang et al., 2022). The specific materials chosen for this study are detailed in Table 3, where each entry corresponds to a particular emotional state aimed to be induced.

**TABLE 3:** Emotion inducing materials selected.

Source Movie	Duration(s)	Arousal	Valence	Emotion	Scene description
—	192	3.96 ± 1.72	5.21 ± 1.34	Calmness	Daily life of a family
Up	67	6.51 ± 1.75	6.87 ± 1.39	Positive	Carl—a shy, quiet boy—meets the energetic Ellie
The Truman Show	60	5.8 ± 1.88	6.29 ± 1.43	Positive	Truman and his lover go to the beach for a romantic evening
Wall-E	93	6.01 ± 1.86	7.26 ± 1.36	Positive	all-E and Eve spend a romantic night together
Gandhi	123	6.15 ± 1.91	3.64 ± 1.23	Negative	Indian attorney gets thrown out of a first-class train compartment
My Bodyguard	101	5.32 ± 2.12	3.48 ± 1.31	Negative	Group of thugs provoke a teenager
The Shining	78	7.41 ± 1.62	2.93 ± 1.46	Negative	A child enters hotel room searching for his mom
Black Swan	62	8.22 ± 1.14	2.38 ± 1.81	Negative	A woman notices paranormal activity around her
My Girl	66	6.39 ± 1.58	3.07 ± 1.4	Negative	A young girl cries at her friend’s funeral
Bambi	166	6.19 ± 1.88	3.48 ± 1.36	Negative	The fawn Bambi’s mother is killed by a deer hunter

The experimental protocol for this dataset was granted approval by the Medical Ethics Committee of the Department of Psychology and Behavioral Sciences at Zhejiang University, as evidenced by the ethical review document (Zhejiang University Psychological Ethics Review [2022] No. 059). A total of 192 students from Zhejiang University were initially recruited to partake in the study. Eligibility criteria for participants included having normal vision, hearing, and perception abilities, as well as being free from any physical or psychological conditions that could potentially influence emotional responses. Throughout the experimental process, stringent quality control measures were implemented. Regrettably, the dataset was compromised due to several factors: (1) instrumental operational errors resulted in the unsuccessful acquisition of 12 cases; (2) 4 cases were excluded due to participants’ personal reasons; and (3) preliminary quality assessment led to the elimination of 19 cases owing to suboptimal physiological signal acquisition. This data attrition, while regrettable, was necessary to maintain the integrity and reliability of the study. Consequently, the dataset was refined to include data and relevant information from 157 participants who met the criteria, comprising 96 females and 61 males.

**FIGURE 5:**
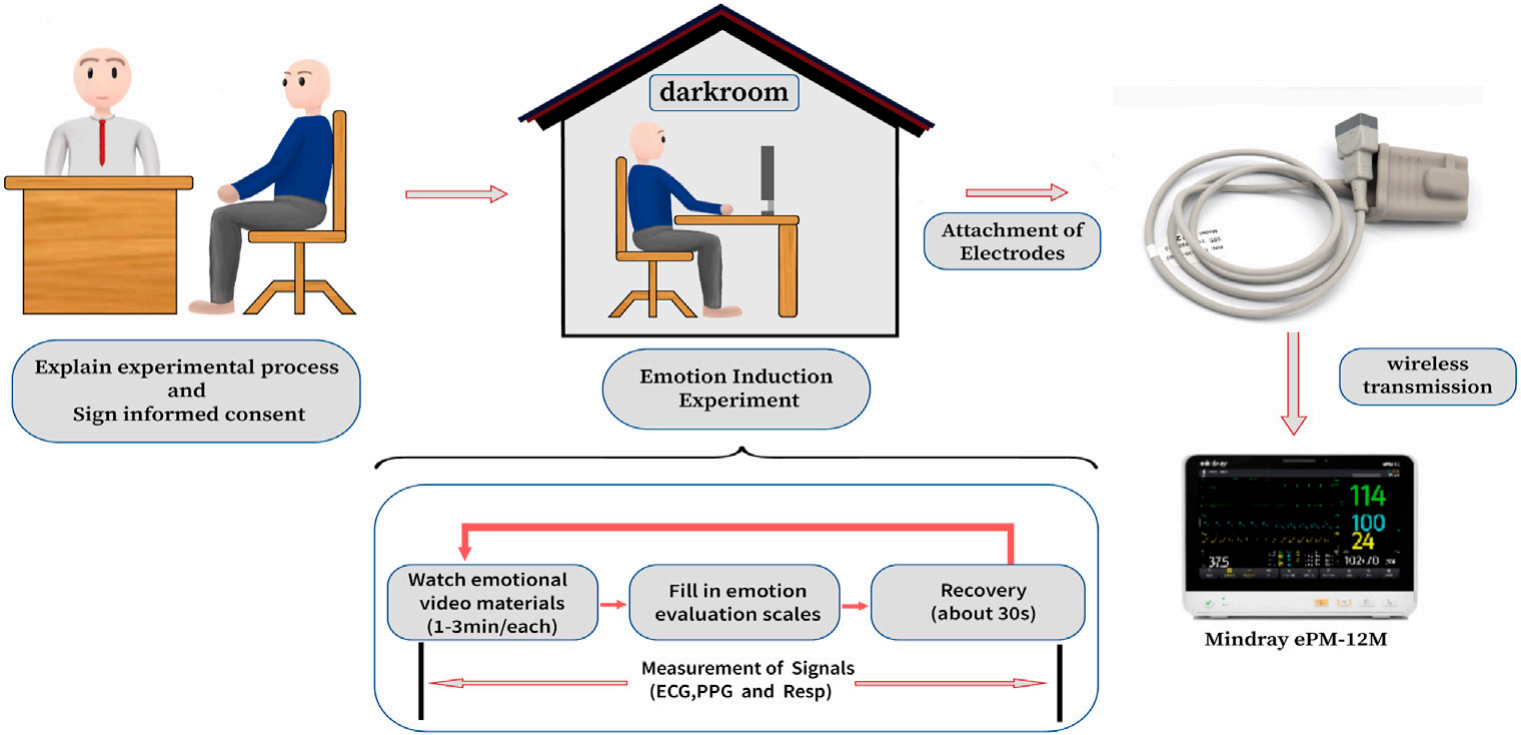
Experimental procedures.

The comprehensive experimental protocol for each participant was conducted in a dimly lit room, ensuring minimal interference from ambient light sources, with the exception of the computer display screen, as depicted in Figure 5. Prior to the commencement of the experiment, participants were provided with a comprehensive briefing on the experimental procedures and necessary precautions. They were required to sign an informed consent form, signifying their voluntary agreement to participate in the study. Subsequently, each participant was outfitted with a photoelectric sensor on their left index finger for the acquisition of PPG signals, as well as electrodes to capture additional physiological signals. The PPG signals were recorded using a physiological signal monitor (ePM-12M, Mindray, China), which operated at a sampling rate of 125 Hz. The video stimuli were presented on a computer screen positioned at a distance ranging from 0.5 to 1 m from the participant, allowing them to view the content from their most comfortable seated position. Throughout the experiment, each participant was exposed to a total of 8 distinct video materials designed to elicit various emotional responses. Upon completion of each video segment, participants were prompted to complete the Self-Assessment Manikin (SAM) Emotion Scale, followed by a brief respite. The sequence of video presentation and scale assessment was orchestrated by a specially designed experimental program, enabling participants to independently execute all steps of the experimental process until its conclusion.

PPG signals are among the most accessible physiological signals for collection; however, they are susceptible to various sources of noise and interference that can complicate the acquisition process. Despite the implementation of hardware-level denoising techniques, the integrity of PPG signals can still be compromised by factors such as respiration, bodily movement, and issues related to data transmission. To ensure the acquisition of high-fidelity PPG signals, it is imperative to employ additional processing measures. In the context of this study, all PPG recordings underwent a series of processing techniques aimed at enhancing signal quality.

Firstly, the 1–20 Hz bandpass filter was used to remove most of the noise and interference caused by breathing, body movements, and other factors, thereby focusing the signal on high-density information regions. Due to the presence of high-frequency disturbances, each PPG record ***y*** was smoothed by Formula 1 and the result was denoted as ***y***_***1***_, where ***x***_***i***_ represented the sampling time of each point, ***i*** represented the sequence number of the data points, 3 ≤ ***i*** ≤ ***n***-1, and ***n*** was the number of data points in the record.

$$\left\{\begin{array}{l} y_{1}\left(x_{1}\right)=y\left(x_{1}\right)\\ y_{1}\left(x_{2}\right)=\frac{y\left(x_{1}\right)+y\left(x_{2}\right)+y\left(x_{3}\right)}{3}\\ y_{1}\left(x_{i}\right)=\frac{y\left(x_{i-2}\right)+y\left(x_{i-1}\right)+y\left(x_{i}\right)+y\left(x_{i+1}\right)+y\left(x_{i+2}\right)}{5}\\ y_{1}\left(x_{n-1}\right)=\frac{y\left(x_{n-2}\right)+y\left(x_{n-1}\right)+y\left(x_{n}\right)}{3}\\ y_{1}\left(x_{n}\right)=y\left(x_{n}\right) \end{array}\right.$$ (1)

**FIGURE 6:**
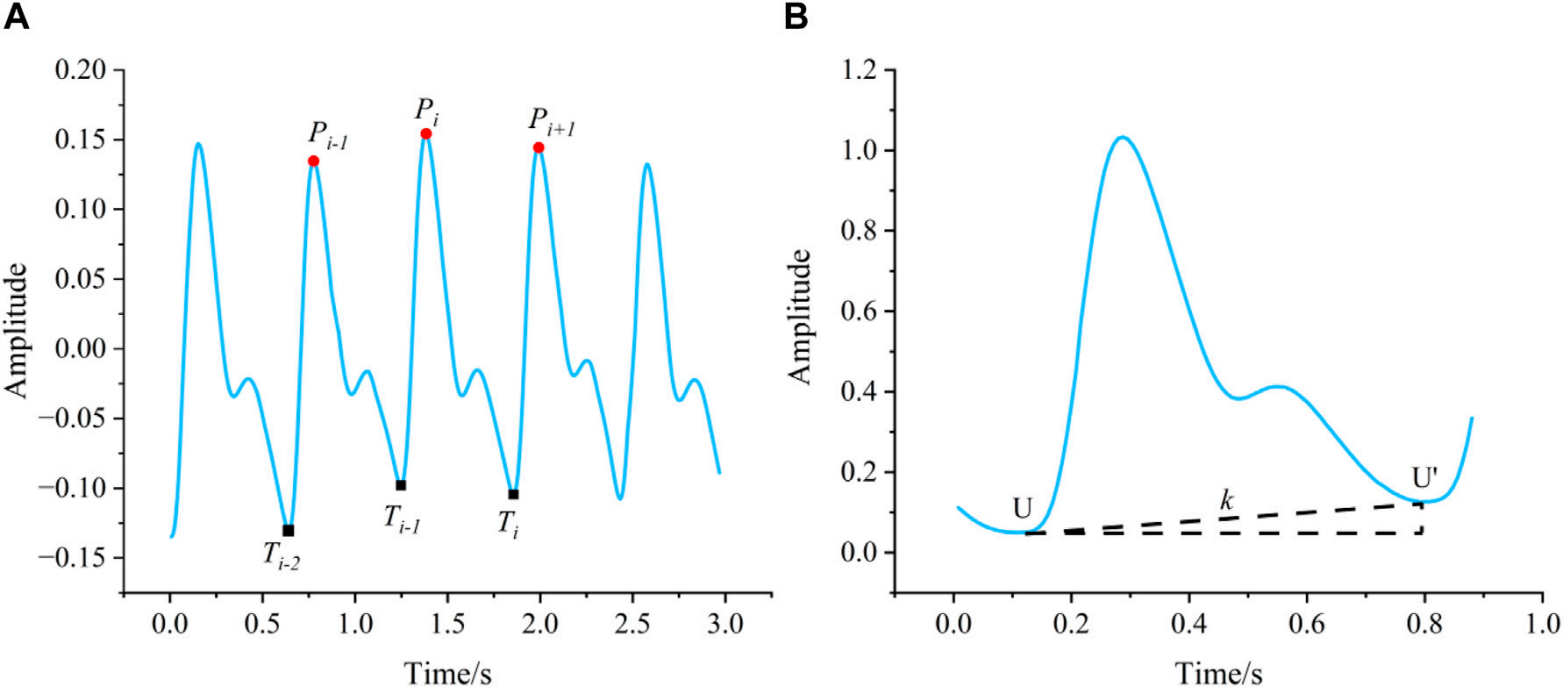
**(A)** The method “moving-pane.” **(B)** The method removing baseline drift.

Then, the method named “moving-pane” was used to identify fiducial points of each PPG record. This method created a pane with a specific width and moved it along the timeline of the PPG record. All maximum points in the pane and minimum points between every two maximum points were recorded during the movement and the maximum points that do not meet the following rules were excluded, as shown in Figure 6A: 1) The distance between this point ***P***_***i***_ and ***P***_***i-1***_ less than 0.6s 2) The amplitude difference between this point ***p***_***i***_ and ***T***_***i-1***_ less than 0.5 times the amplitude difference between ***P***_***i-1***_ and ***T***_***i-2***_, or the amplitude difference between ***p***_***i***_ and ***T***_***i-1***_ less than 0.5 times the amplitude difference between ***p***_***i+1***_ and ***T***_***i.***_

Following the application of the “moving-pane” method, a meticulous manual review was performed to identify and retain the extremum points within the PPG record, which correspond to the peaks and troughs that serve as the fiducial markers of the waveform. This process is crucial for the accurate characterization of the PPG signal’s morphology. Subsequently, to address the issue of baseline drift that can distort the PPG signal, the method illustrated in Figure 6B and encapsulated by Formula 2 was employed. This technique effectively removes the baseline wander, ensuring that the origin of all individual PPG waveforms is recalibrated to zero. This normalization is essential for the consistent analysis and comparison of PPG signals across different recordings.

$$\left\{\begin{array}{l} k=\frac{y\left(x_{U}\right)-y\left(x_{U^{\prime }}\right)}{x_{U}-x_{U^{\prime }}}\\ y^{\prime }\left(x_{i}\right)=y\left(x_{i}\right)-k*\left(x_{i}-x_{U}\right) \end{array}\right.$$ (2)

In the concluding phase of signal processing, the z-score normalization technique was applied to eliminate the variability in the scale of each PPG record. This standardization procedure ensures that all records are brought onto a common scale, facilitating a unified analysis. The aforementioned processing methodologies were executed using a combination of self-devised algorithms and the scipy package (Virtanen et al., 2020), which is a fundamental component of the Python ecosystem for scientific computing. Post-processing, each PPG record was meticulously segmented in accordance with the distinct emotional stimuli that served as the triggers. Specifically, the records were divided into segments every 20 s, with each segment annotated to reflect the predominant emotional state during that interval.

### 2.3 Features extraction and emotion analysis

Following the preprocessing of PPG signals, the data were segmented according to different emotional stimuli protocols. The continuous recordings were subsequently divided into 20-s epochs, with each epoch being annotated with corresponding emotional labels. This segmentation procedure yielded a total of 2,474 epochs for low arousal states, 5,560 epochs for high arousal states, 3,229 epochs for low valence states, and 4,805 epochs for high valence states, thereby establishing a comprehensive dataset for emotional state classification.

For each 20-s epoch, nine frequency-domain features (as specified in Table 3) were extracted. To account for inter-individual variability and other potential confounding factors, feature normalization was performed using a z-score-like transformation according to Equation 3. This standardization procedure ensures comparability across different subjects while preserving the relative distribution characteristics of the extracted features.

$$F_{remove}=\left(F_{emotion}-F_{mean}\right)/F_{std}$$ (3)

Among them, for specific feature ***F*** and a certain subject ***S***, ***F***_***mean***_ and ***F***_***std***_ were the average and standard deviation of feature ***F*** for all sections of subject ***S***, ***F***_***emotion***_ was the value of feature ***F*** before processing for a specific emotional section of subject ***S***, and ***F***_***remove***_ was the processed feature value.

The Mann Whitney U-test (Rosner and Grove, 1999), a non-parametric statistical method, serves as a robust tool for assessing significant differences between two independent datasets, especially when the data does not meet the assumptions of parametric tests. In this study, the U-test, facilitated by Python’s SciPy library (Virtanen et al., 2020), was employed to scrutinize the variability in the newly extracted PPG frequency-domain features across different emotional dimensions, specifically comparing the high and low arousal states, as well as the high and low valence states. The preliminary evaluation of the emotion-discriminative capability of the extracted PPG frequency-domain features was conducted through two complementary approaches: (1) statistical analysis using p-values derived from the two-tailed U-test, and (2) comparative examination of feature distribution patterns across different emotional states. This dual-method assessment framework provides robust evidence for evaluating the effectiveness of the proposed features in emotion differentiation.

Subsequently, based on the preliminary analysis, the identified emotion-discriminative features were utilized to construct a machine learning model for emotion recognition using Support Vector Machines (SVM). In this study, the SVM was implemented using the Scikit-learn algorithm package in Python (Fabian et al., 2011). To effectively partition the dataset, the *train_test_split* function from the Scikit-learn package was utilized, segregating the feature set into training and testing subsets with a ratio of 7:3. Given the modest size of the dataset, traditional cross-validation could potentially result in overfitting. Consequently, the study opted for a leave-one-point-out method for model training, a technique that iteratively excludes a single data point from the training process. This approach was iterated 100 times, ensuring a comprehensive assessment of the model’s performance (Ma et al., 2023). The Area Under the Curve (AUC) of the Receiver Operating Characteristic (ROC) curve was computed for each iteration. The median AUC value, derived from the 100 models, was adopted as the representative performance metric for the SVM model.

ROC curves (Rodellar-Biarge et al., 2015) were employed to evaluate and visualize the discriminatory power of the features in identifying distinct emotional states. The model’s predictive accuracy, the AUCs for the ROC curves, and the precision metric were computed to quantitatively assess the model’s efficacy in recognizing emotions.

### 2.4 Feature comparison and cross dataset validation

To further validate the effectiveness of the extracted PPG frequency-domain features while maintaining the exclusive use of PPG as the sole physiological signal, we additionally extracted two well-established feature sets that have been extensively validated by numerous researchers for emotion recognition: pulse rate variability (PRV) features and PPG morphological features, as detailed in Table 4. Following the same analytical protocol applied to the frequency-domain features, these comparative features underwent preliminary screening before being utilized to construct SVM-based machine learning models, thereby obtaining their respective emotion recognition accuracy metrics for systematic comparison. Furthermore, to investigate the underlying relationships among different feature sets, we conducted correlation analysis between the PPG frequency-domain features and the two additional feature groups. This analysis facilitated the development of an integrated feature set through optimal feature fusion, potentially enhancing the overall emotion recognition performance.

**TABLE 4:** Features for comparison.

Category	Features
PRV	MeanNN, SDNN, RMSNN, MedianNN, RDNN, IQRNN, CVNN, SDSD, RMSSD, CVSD, pNN20, ApEn, FuzzyEn, LZC
Morphological Features	Amp_Diff, Interval_Rise, Interval_Drop, Slope_Rise, Slope_Max_Rise, Slope_Drop, Slope_Min_Drop, Area_Rise, Area_Drop, Area_Total, Area_Rise_rate, Area_Drop_rate, Area_Total_rate, Area_RD_rate

To ascertain the efficacy and generalizability of the methodologies and features delineated in this research, an expanded dataset of PPG recordings was sourced from the DEAP database, which is publicly accessible. The DEAP dataset (Koelstra et al., 2012) serves as a multimodal repository for affective analysis, encompassing physiological signal recordings from 32 participants exposed to 40 distinct video stimuli. For the purpose of this analysis, PPG recordings were exclusively selected. Consistent with the methodology applied to the aforementioned dataset, participants were prompted to rate the arousal, valence, and additional pertinent attributes of each video stimulus. The models established in the preceding section were then applied to discern the emotional states associated with the DEAP dataset entries. Subsequently, the derived accuracy metrics were utilized to assess the models’ performance in emotion recognition and their adaptability across different datasets.

## 3 Results

**FIGURE 7:**
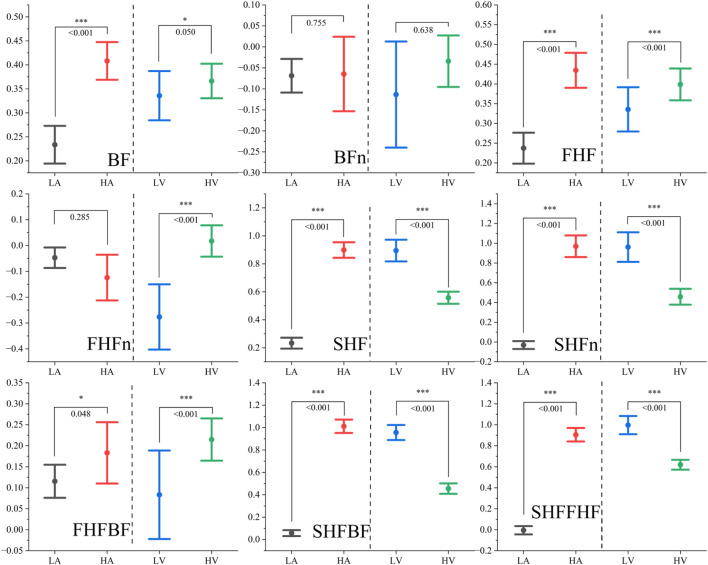
The distribution of Peaks-based frequency-domain features under different emotional states. Note: *represents p < 0.05, ** represents p < 0.01, *** represents p < 0.001.

Figure 7 presents the distribution patterns of the nine extracted PPG frequency-domain features across different emotional states, categorized by high/low arousal and high/low valence. The corresponding p-values derived from U-tests, which indicate the statistical significance of differences between high and low arousal states as well as between high and low valence states, are also displayed. The analysis reveals distinct patterns of feature variations in response to different emotional dimensions.

Regarding arousal levels, we observe synchronous increases in both the fundamental frequency and the first harmonic frequency, while the ratio-based features remain relatively stable. Notably, features associated with the second harmonic frequency demonstrate significant enhancement, indirectly reflecting the overall increase in total power during heightened arousal states. This observed phenomenon can be primarily attributed to the physiological correlates of increased peripheral vascular resistance and enhanced blood flow intensity. These physiological changes are consistent with the characteristic manifestations of heightened arousal states, which typically involve muscle tension, vasoconstriction, and intensified cardiac activity resulting from emotional excitation (Shu et al., 2018).

In contrast, valence levels exhibit a different pattern of influence: while both the fundamental frequency and the first harmonic frequency show moderate increases (with the first harmonic demonstrating more pronounced enhancement), the second harmonic frequency displays a marked decrease. Interestingly, the total power remains relatively unaffected by changes in valence. This observation is strongly associated with the fundamental nature of valence as a psychophysiological dimension that primarily reflects the distinction between positive and negative affective states (Gendolla and Krusken, 2001; Fairclough et al., 2014).

**FIGURE 8:**
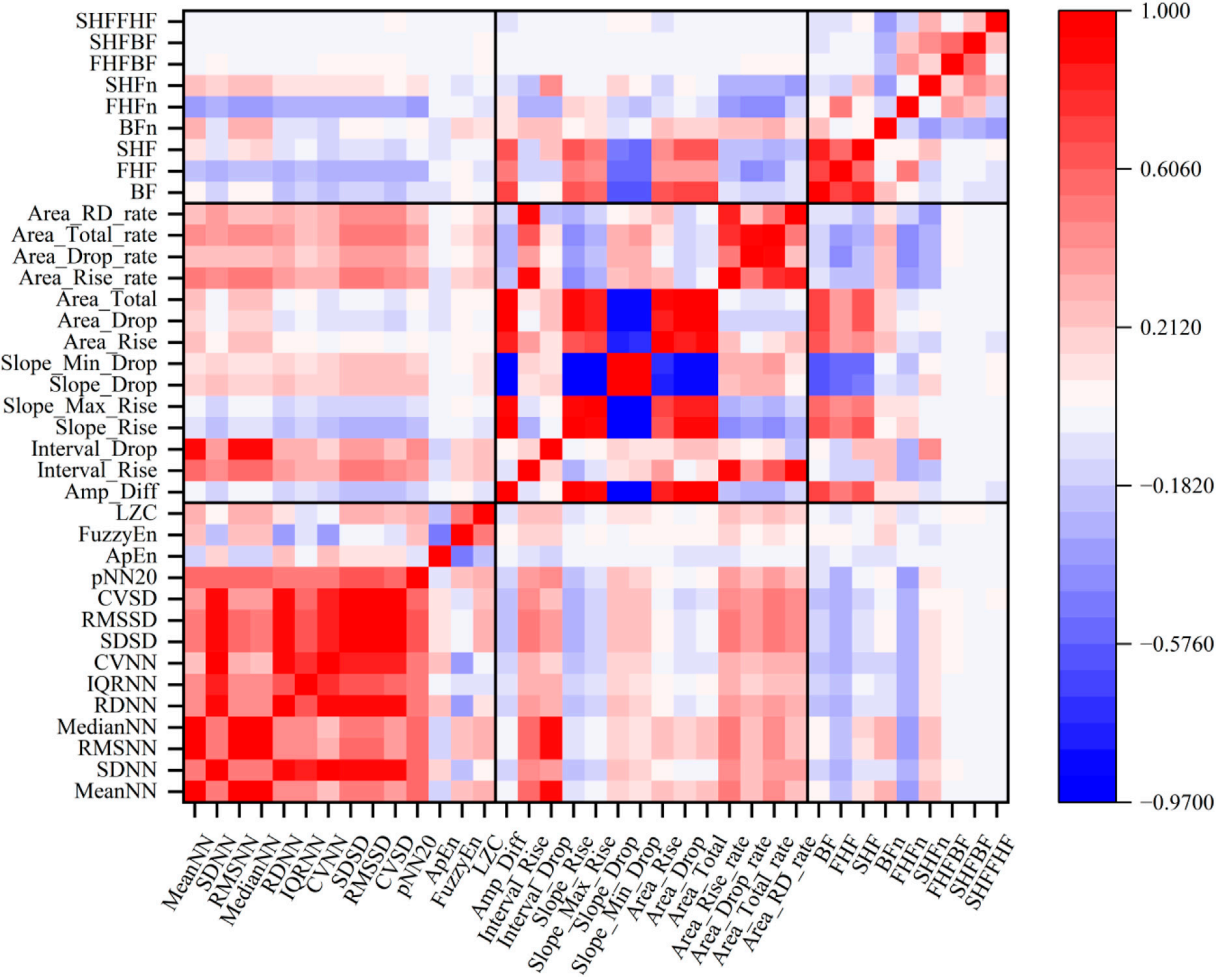
Results of intra-group and inter-group orrelation analyses.

The analysis clearly demonstrates that the PPG frequency-domain features exhibit significant sensitivity to both arousal and valence variations, as evidenced by their systematic changes corresponding to different emotional states. To further evaluate the effectiveness of these features in emotion recognition, we conducted a comparative analysis with two well-established feature sets: PRV features and PPG morphological features. Figure 8 presents the results of intra-group and inter-group correlation analyses among these three feature sets.

The correlation matrix reveals several important patterns: (1) The PPG frequency-domain features maintain relatively low intra-group correlations, suggesting their complementary nature in capturing different aspects of emotional states. (2) Moderate correlations between frequency-domain features and morphological features indicate that the spectral information partially reflects certain morphological characteristics of PPG signals. (3) Both PRV and morphological feature sets exhibit substantially higher intra-group correlations compared to the frequency-domain features, indicating greater redundancy within these conventional feature sets. This comparative analysis suggests that the proposed frequency-domain features offer a more diverse and potentially more efficient representation of emotional states.

**FIGURE 9:**
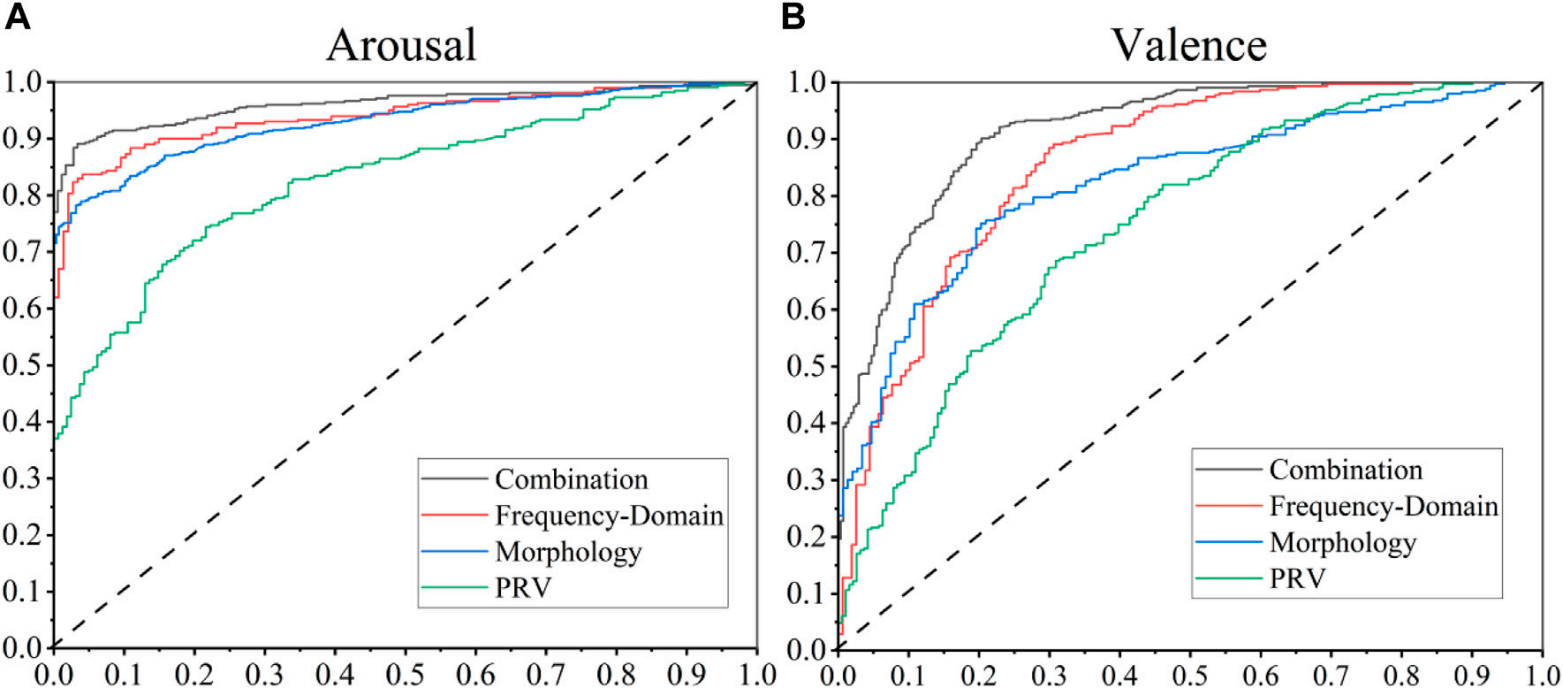
ROC curves for distinguishing **(A)** arousal levels; **(B)** valence levels.

The comparative results of feature performance are presented in Table 5 and Figure 9. Among the three feature sets, the proposed PPG frequency-domain features demonstrated superior performance in machine learning models, achieving an accuracy of 87.5% in arousal classification and 81.4% in valence classification. The higher accuracy in arousal classification aligns with the more pronounced feature distribution differences observed in arousal states, as previously discussed. The PPG morphological features also showed reasonable discriminative capability, while the PRV features exhibited relatively poor performance. The suboptimal performance of PRV features may be attributed to two potential factors: (1) The exclusion of traditionally effective features such as LF and HF components, which could not be accurately computed due to the short duration (20s) of individual epochs; (2) The high intra-feature correlation within the PRV feature set, resulting in substantial redundancy despite the large number of features, effectively reducing the dimensionality of useful information.

**TABLE 5:** The accuracy infornmation of models.

Features	Dataset	Emotion	Accuracy	AUC	Precision	Sensitivity	Specificity	F1-score
Combination	Mine	LA-HA	91.0%	0.961	0.972	0.898	0.939	0.933
Frequency-Domain			87.5%	0.940	0.980	0.831	0.965	0.899
Morphology			84.4%	0.929	0.978	0.784	0.965	0.871
PRV			75.7%	0.827	0.873	0.747	0.778	0.805
Combination		LV-HV	85.9%	0.916	0.881	0.893	0.804	0.887
Frequency-Domain			81.4%	0.852	0.899	0.814	0.814	0.854
Morphology			75.9%	0.824	0.895	0.743	0.797	0.812
PRV			71.1%	0.747	0.757	0.799	0.560	0.778
Combination	DEAP	LA-HA	79.3%	0.784	0.771	0.915	0.628	0.836
Frequency-Domain			73.5%	0.741	0.709	0.881	0.555	0.786
Morphology			72.6%	0.739	0.698	0.880	0.540	0.778
PRV			70.6%	0.714	0.682	0.855	0.530	0.759
Combination		LV-HV	75.9%	0.762	0.738	0.882	0.602	0.804
Frequency-Domain			70.9%	0.707	0.694	0.848	0.539	0.763
Morphology			71.5%	0.718	0.686	0.872	0.530	0.768
PRV			69.5%	0.699	0.676	0.842	0.520	0.750

To further validate the generalizability of our methodology, we replicated the analytical procedure on PPG signals from the DEAP dataset, maintaining identical processing pipelines and model construction approaches. The comparative results, as presented in Table 5, demonstrate remarkable consistency with our proprietary dataset findings, though with marginally reduced classification accuracy in the DEAP dataset. This performance variation can be attributed to multiple factors, including the use of default SVM parameters without specific optimization and inherent differences in emotional elicitation protocols between studies.

Capitalizing on the observed low inter-group feature correlations, we implemented a comprehensive feature fusion strategy. This integrated approach yielded exceptional emotion recognition performance, achieving accuracy rates surpassing 90% on our proprietary dataset. These findings not only confirm the standalone efficacy of the proposed PPG frequency-domain features in emotion recognition but also establish their crucial role as a fundamental component in advanced emotion recognition systems. The features’ unique complementary characteristics make them an indispensable element in the pursuit of enhanced recognition performance, serving as a critical piece in the development of more sophisticated emotion classification frameworks.

## 4 Discussion

This study employs a physiological model-based simulation approach to systematically analyze the frequency-domain components of PPG signals and extract their essential characteristics. Through this comprehensive investigation, we examine the efficacy of these frequency-domain features in effectively discriminating emotional states. Furthermore, the research elucidates the intricate relationships between physiological parameters and emotional states, as well as the connections between PPG frequency-domain features and emotional responses, thereby providing a deeper understanding of the psychophysiological mechanisms underlying emotion recognition.

Through comprehensive investigation of PPG frequency-domain features, we have identified significant correlations between these features and various physiological parameters, including peripheral vascular resistance, blood flow inertia, and vascular compliance. Moreover, these features demonstrate remarkable sensitivity to variations in both arousal and valence levels, thereby establishing a crucial tripartite relationship among physiological parameters, PPG frequency-domain characteristics, and emotional states. These findings provide valuable psychophysiological foundations and references for subsequent emotion recognition analyses based on PPG frequency-domain features.

An intriguing observation from both our proprietary dataset and the DEAP dataset reveals that PPG frequency-domain features exhibit greater sensitivity to arousal levels compared to valence. This phenomenon may be attributed to the more pronounced cardiovascular changes associated with emotional intensity (arousal) rather than emotional polarity (positive/negative valence), suggesting that the autonomic nervous system’s response to emotional arousal might be more substantial and detectable through PPG analysis.

In comparison with existing studies, as summarized in Table 6, research utilizing PPG signals for emotion recognition remains relatively scarce, with even fewer studies employing PPG as the primary or exclusive physiological modality. When considering variations in emotional elicitation materials and differing analytical focuses across studies, our research demonstrates competitive emotion recognition accuracy, positioning itself within the upper-middle range of existing literature. This represents a significant achievement in the field. Notably, findings from other researchers corroborate our observations regarding the suboptimal performance of PRV features and the relatively better performance of morphological features. However, what distinguishes our study is the establishment of a comprehensive theoretical framework that bridges physiological parameters, PPG frequency-domain features, and emotional states. This tripartite model provides robust psychophysiological evidence supporting the use of PPG frequency-domain features for emotion recognition, thereby advancing our understanding of the underlying mechanisms and offering a solid theoretical foundation for future research in this domain.

**TABLE 6:** Comparisons with other studies.

References	Signals	Scourse	Features (PPG) or method	Accuracy or result
Lee et al. (2019)	PPG	DEAP	Features extracted through CNN	Valence: 75.3% Arousal: 76.2%
Choi and Kim (2018)	EEG, PPG, Video	DEAP	LSTM	Valence: 78% Arousal: 74.65%
Paul et al. (2024)	PPG	DEAP	A New Morphological Feature	Special emotions: 97.78%
Beckmann et al. (2019)	PPG	Experiment	Pulse Transit Time (PTT)	Significant Effectiveness
Li et al. (2017)	PPG	Experiment	PRV, Morphological Features	1) Morphological features superior to PRV features 2) Frequency domain features (LF, etc.) superior to time domain features (SDNN, etc.) in PRV
Wang and Yu (2021)	PPG	Experiment	Deap Learning	Two classes: 89.15% Four classes: 84.70%
Kang and Kim (2022)	PPG, GSR	DEAP, MERTI-Apps	Deap Learning	Valence: 73.49% Arousal: 77.87%
Yang et al. (2024)	PPG, GSR, EEG, Viedo	Experiment	PRV, Morphological Features	Positive-Negative: 80.96%
Lee et al. (2020)	PPG	DEAP	PRV, Features extracted through CNN	Valence: 82.1% Arousal: 80.9%
This Study	PPG	Experiment, DEAP	Frequency Domain Features, PRV, Morphological Features	(Experiment) Valence: 91.0% (Experiment) Arousal: 85.9% (DEAP) Valence: 75.9% (DEAP) Arousal: 79.3%

Nevertheless, this study is subject to several limitations that warrant consideration. First, while we have established a tripartite framework connecting physiological parameters, PPG frequency-domain features, and emotional states, the current analysis primarily demonstrates their strong associations rather than establishing precise quantitative correlations. This limitation highlights the need for more sophisticated modeling approaches to quantify these relationships. Secondly, the selection of emotional elicitation materials presents inherent challenges. The material-specific characteristics sometimes exert a more substantial influence on the extracted features than the emotional states themselves. Additionally, the duration of stimulus materials significantly impacts feature selection and interpretation, a methodological concern that persists across numerous studies in this field. Finally, our research primarily focused on feature analysis and interpretation, with relatively less emphasis on optimization for classification accuracy. This methodological orientation, while providing valuable insights into feature characteristics, has resulted in classification performance that, while respectable, leaves room for improvement. Future studies should aim to strike a better balance between feature exploration and recognition performance optimization.

## 5 Conclusion

This study employs a physiology model-based simulation approach to systematically analyze the frequency-domain components of PPG signals and extract their essential characteristics. Through comprehensive investigation, we examine the efficacy of these frequency-domain features in effectively discriminating emotional states. Furthermore, the research elucidates the intricate relationships between physiological parameters, frequency-domain characteristics, and emotional states, thereby providing deeper insights into the psychophysiological mechanisms underlying emotion recognition. These findings establish a solid theoretical foundation and offer valuable references for subsequent research in this field.

## Data Availability

The raw data supporting the conclusions of this article will be made available by the authors, without undue reservation.
