# Supplementary material for: An emotion recognition method based on frequency-domain features of PPG
Source: Front Physiol. 2025 Feb 25;16:1486763. doi: 10.3389/fphys.2025.1486763 (PMC11893849; doi:10.3389/fphys.2025.1486763)
Supplement: Supplementary file 1 [file Table1.docx]

Supplementary Material

# Supplementary Data

The following table shows the fitting expression between PPG frequency domain characteristics and physiological parameters used in the article. The relationship between the two is obtained through polynomial fitting, which includes invariance, linear variation, and curve variation. The fitting expression is in the form of the following expression.

$$f=a_{1}x^{3}+a_{2}x^{2}+a_{3}x+a_{4}$$

| Physiological Parameters | Features | a_1_ | a_2_ | a_3_ | a_4_ |
| --- | --- | --- | --- | --- | --- |
| R | BF | 3.30 | -10.21 | 10.11 | 197.75 |
| R | FHF | 39.02 | -132.97 | 153.38 | -6.97 |
| R | SHF | -1.30 | 3.16 | 1.16 | -0.19 |
| R | BFn | -0.15 | 0.52 | -0.59 | 1.01 |
| R | FHFn | 0.16 | -0.52 | 0.58 | -0.01 |
| R | SHFn | 0.00 | 0.01 | 0.01 | 0.00 |
| R | FHFBF | 0.19 | -0.66 | 0.76 | -0.03 |
| R | SHFBF | -0.01 | 0.02 | 0.01 | 0.00 |
| R | SHFFHF | -0.02 | 0.05 | 0.02 | 0.00 |
| L | BF | -1935000.23 | 151313.49 | 1296.90 | 156.03 |
| L | FHF | -26965861.74 | 1279026.72 | -13516.65 | 62.25 |
| L | SHF | 4889578.21 | -240066.65 | 3429.67 | -11.43 |
| L | BFn | 63868.31 | -2768.03 | 25.89 | 0.80 |
| L | FHFn | -75887.13 | 3277.98 | -30.80 | 0.19 |
| L | SHFn | 22170.44 | -1073.07 | 15.02 | -0.05 |
| L | FHFBF | -127364.22 | 5666.35 | -57.80 | 0.30 |
| L | SHFBF | 26497.67 | -1289.52 | 18.19 | -0.06 |
| L | SHFFHF | 147634.23 | -6930.89 | 92.45 | -0.26 |
| C1 | BF | -1389.77 | 5090.29 | -6040.28 | 2452.05 |
| C1 | FHF | -156.72 | 609.05 | -795.98 | 381.13 |
| C1 | SHF | -2.63 | 33.03 | -75.38 | 46.75 |
| C1 | BFn | 0.01 | -0.04 | 0.02 | 0.80 |
| C1 | FHFn | 0.04 | -0.17 | 0.22 | 0.10 |
| C1 | SHFn | 0.02 | -0.06 | 0.03 | 0.02 |
| C1 | FHFBF | 0.05 | -0.20 | 0.28 | 0.13 |
| C1 | SHFBF | 0.03 | -0.08 | 0.04 | 0.02 |
| C1 | SHFFHF | 0.11 | -0.25 | 0.03 | 0.15 |
| C2 | BF | 1.41 | 17.69 | -69.84 | 212.13 |
| C2 | FHF | 20084.78 | -6517.69 | 881.35 | -11.18 |
| C2 | SHF | 1319.58 | -797.98 | 137.68 | -4.73 |
| C2 | BFn | -17.72 | 3.07 | -0.88 | 0.94 |
| C2 | FHFn | 12.42 | 0.03 | 0.38 | 0.07 |
| C2 | SHFn | 5.60 | -3.36 | 0.57 | -0.02 |
| C2 | FHFBF | 107.18 | -35.00 | 4.70 | -0.07 |
| C2 | SHFBF | 6.44 | -3.91 | 0.68 | -0.02 |
| C2 | SHFFHF | 40.33 | -22.78 | 3.56 | -0.10 |
| q0 | BF | 0.00 | 0.00 | 0.00 | 0.00 |
| q0 | FHF | 0.00 | 0.00 | 0.00 | 0.00 |
| q0 | SHF | 0.00 | 0.00 | 0.00 | 0.00 |
| q0 | BFn | 0.00 | 0.00 | 0.00 | 0.79 |
| q0 | FHFn | 0.00 | 0.00 | 0.00 | 0.20 |
| q0 | SHFn | 0.00 | 0.00 | 0.00 | 0.01 |
| q0 | FHFBF | 0.00 | 0.00 | 0.00 | 0.25 |
| q0 | SHFBF | 0.00 | 0.00 | 0.00 | 0.01 |
| q0 | SHFFHF | 0.00 | 0.00 | 0.00 | 0.04 |
| Td | BF | -411.84 | 1360.38 | -1008.23 | 353.22 |
| Td | FHF | 1283.64 | -3296.45 | 2686.21 | -636.09 |
| Td | SHF | -74.07 | 166.47 | -110.37 | 22.51 |
| Td | BFn | -5.52 | 14.27 | -11.58 | 3.71 |
| Td | FHFn | 5.72 | -14.75 | 11.90 | -2.77 |
| Td | SHFn | -0.25 | 0.54 | -0.35 | 0.07 |
| Td | FHFBF | 9.37 | -23.97 | 19.15 | -4.46 |
| Td | SHFBF | -0.29 | 0.62 | -0.39 | 0.08 |
| Td | SHFFHF | -1.84 | 4.18 | -2.82 | 0.59 |
| Ts | BF | -2673.64 | 2561.44 | -99.31 | 4.20 |
| Ts | FHF | 2863.95 | -4314.60 | 1902.40 | -204.35 |
| Ts | SHF | 1082.80 | -682.71 | -45.96 | 60.24 |
| Ts | BFn | -6.37 | 5.79 | -0.28 | 0.39 |
| Ts | FHFn | 8.83 | -11.74 | 4.15 | -0.15 |
| Ts | SHFn | -1.85 | 5.22 | -3.59 | 0.72 |
| Ts | FHFBF | 6.56 | -6.13 | 0.09 | 0.77 |
| Ts | SHFBF | -10.36 | 16.95 | -8.97 | 1.55 |
| Ts | SHFFHF | 31.55 | -27.94 | 5.88 | 0.14 |

# Supplementary Figures and Tables

## Supplementary Figures

**
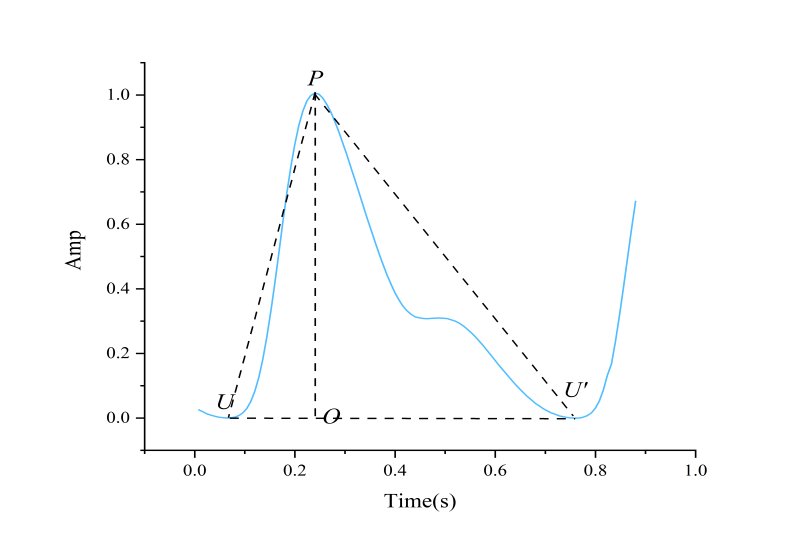
**

**Supplementary Figure 1.** Schematic diagram of PPG morphological characteristics

## Supplementary Tables

| ***Features*** | ***Description*** |
| --- | --- |
| MeanNN | Average value of NN interval sequence |
| SDNN | Standard deviation of NN interval sequence |
| RMSNN | Root mean square value of NN interval sequence |
| MedianNN | Median of NN interval sequence |
| RDNN | Range of NN interval sequences |
| IQRNN | Interquartile distance of NN interval sequence |
| CVNN | Standard deviation of NN intervals divided by the average of NN intervals |
| SDSD | Standard deviation of adjacent NN interval difference sequence |
| RMSSD | Root mean square value of adjacent NN interval difference sequence |
| CVSD | Mean square eradication of the difference between adjacent NN intervals is based on the average of NN intervals |
| pNN20 | The proportion of NN20 to the total number of NN intervals |
| ApEn | ANNroximate Entropy of NN Interval Sequences |
| FuzzyEn | Fuzzy Entropy of NN Interval Sequences |
| LZC | LZ complexity of NN interval sequences |

**Supplementary Table 1.** PRV features and their definitions

| ***Features*** | ***Description*** |
| --- | --- |
| AmpDiff | The amplitude difference between the peak and valley, as shown OP |
| Rise_IntervalDiff | Upward branch time interval, as shown UO |
| Drop_IntervalDiff | Descending branch time interval, as shown OU' |
| Rise_SlopeMean | The average slope of the upward branch, as shown the slope of UP |
| Rise_SlopeMax | The maximum slope of the upward branch, as shown the maximum slope of the UP segment of the curve |
| Drop_SlopeMean | The average slope of the descending branch, as shown the slope of PU' |
| Drop_SlopeMin | The maximum slope of the descending branch, as shown the maximum slope of the PU' segment of the curve |
| AreaRise | The area of the upward branch, as shown in the area enclosed by UO, OP, and the curve UP |
| AreaDrop | The area of the descending branch, as shown in the area enclosed by OU', OP, and the curve PU' |
| AreaTotal | The area of the entire waveform |
| AreaRiseRate | AreaRise/△UOP's area |
| AreaDropRate | AreaDrop/△U'OP's area |
| AreaTotalRate | AreaTotal/△U'PU's area |
| AreaRDRate | AreaRise/AreaDrop |

**Supplementary Table 2.** PPG morphological features and definitions, using Supplementary Figure 1 as a reference
